# Pancreatic Cancer-Derived Small Extracellular Vesicles Remodel Hepatic Pre-Metastatic Niche via Hybrid Epithelial–Mesenchymal States

**DOI:** 10.3390/ijms27125270

**Published:** 2026-06-10

**Authors:** Francesco Balestra, Giorgia Panzetta, Maria De Luca, Federica Rizzi, Anna Ancona, Ilaria Grassi, Roberto Comparelli, Maria Lucia Curri, Gianluigi Giannelli, Nicoletta Depalo, Maria Principia Scavo

**Affiliations:** 1 Laboratory of Molecular Medicine, National Institute of Gastroenterology, IRCCS “S. de Bellis” Research Hospital, Via Turi 27, Castellana Grotte, 70013 Bari, Italy; francesco.balestra@irccsdebellis.it (F.B.); giorgia.panzetta@irccsdebellis.it (G.P.); maria.deluca@irccsdebellis.it (M.D.L.); 2 Institute for Chemical-Physical Processes, Italian National Research Council (IPCF)-CNR SS Bari, Via Orabona, 70125 Bari, Italy; federica.rizzi@cnr.it (F.R.); roberto.comparelli@cnr.it (R.C.); marialucia.curri@uniba.it (M.L.C.); nicoletta.depalo@cnr.it (N.D.); 3 Bari Research Unit, National Interuniversity Consortium of Materials Science and Technology (INSTM), Via Orabona 4, 70126 Bari, Italy; 4 Core Facility Biobank, National Institute of Gastroenterology, IRCCS “S. de Bellis” Research Hospital, Via Turi 27, Castellana Grotte, 70013 Bari, Italy; anna.ancona@irccsdebellis.it; 5 Department of Pathology, National Institute of Gastroenterology, IRCCS “S. de Bellis” Research Hospital, Via Turi 27, Castellana Grotte, 70013 Bari, Italy; ilaria.grassi@irccsdebellis.it; 6 Department of Chemistry, University of Bari, Via Orabona 4, 70125 Bari, Italy; 7 Scientific Direction, National Institute of Gastroenterology, IRCCS “S. de Bellis” Research Hospital, Via Turi 27, Castellana Grotte, 70013 Bari, Italy; gianluigi.giannelli@irccsdebellis.it

**Keywords:** PDAC, small extracellular vesicles, hepatic microenvironment, liver metastasis, pre-metastatic niche

## Abstract

Pancreatic ductal adenocarcinoma frequently metastasises to the liver, although the mechanisms underlying hepatic pre-metastatic niche formation remain unclear. Small extracellular vesicles mediate tumour–host communication and may drive hepatic microenvironment reprogramming. This study investigated the effects of pancreatic ductal adenocarcinoma-derived small extracellular vesicles on extracellular matrix remodelling and epithelial–mesenchymal transition-related plasticity in hepatic cells. Small extracellular vesicles were isolated from pancreatic ductal adenocarcinoma cell lines (MIAPaCa-2, PANC-1) and from the serum of 25 patients, characterized, and administered to hepatic stellate (LX-2) and hepatocyte-like (HEPA-RG) cells. Cell viability and migration were evaluated by functional assays, morphology by scanning electron microscopy, and molecular changes by RT-PCR, Western blotting, and immunofluorescence. In LX-2 cells, small extracellular vesicles exposure increased metabolic activity, adhesion, and migration, while inducing morphological and molecular changes associated with extracellular matrix remodelling, including reduced collagen type I alpha 2 chain, vimentin, and E-cadherin expression. In HEPA-RG cells, viability was minimally affected, whereas migration and EMT-related plasticity were enhanced. Patient-derived small extracellular vesicles induced similar but less pronounced effects. Overall, pancreatic ductal adenocarcinoma-derived small extracellular vesicles induced early hepatic microenvironmental remodelling, supporting a potential role for tumour–liver crosstalk in pre-metastatic niche-associated processes, highlighting tumour–liver crosstalk as a potential therapeutic target.

## 1. Introduction

Pancreatic ductal adenocarcinoma (PDAC) remains one of the deadliest malignancies worldwide, largely due to its aggressive biology, early metastatic dissemination, and resistance to conventional therapies [1]. A key driver of PDAC progression is the epithelial–mesenchymal transition (EMT), a dynamic and plastic process through which epithelial cancer cells acquire mesenchymal traits, enhancing migratory and invasive capabilities [2].

Notably, EMT is not a binary switch, but a continuum of intermediate states characterized by variable combinations of epithelial and mesenchymal features [3,4], regulated by complex signalling networks within the tumour microenvironment (TME) that involve dynamic interactions between tumour cells and stromal components, including cancer-associated fibroblasts and the extracellular matrix (ECM). These interactions support tumour progression by promoting desmoplasia, immune suppression, and therapeutic resistance [5,6,7]. Collectively, these processes highlight the central role of TME-driven cellular plasticity in PDAC and support the targeting of EMT-related pathways and stromal interactions as potential therapeutic strategies.

Extracellular vesicles (EVs) are nano-sized, lipid bilayer-enclosed particles secreted by virtually all cell types and have emerged as key mediators of intercellular communication within the tumour microenvironment. According to the MISEV2023 guidelines, EVs are broadly classified by size into small EVs (sEVs, <200 nm) and large EVs (lEVs, >200 nm) [8]. Among these, sEVs have gained particular attention due to their ability to transfer bioactive cargo, thereby promoting tumour progression, immune evasion, and pre-metastatic niche formation [9,10]. In PDAC, sEVs have been shown to modulate processes such as oxidative stress responses and fibrogenesis in both cancer and stromal cells, contributing to the establishment of a desmoplastic and pro-tumorigenic microenvironment [11].

The liver represents the most frequent site of PDAC metastasis, reflecting a preferential tumour–organ axis [12], sEVs actively interact with resident hepatic cell populations, including hepatocytes and hepatic stellate cells (HSCs) [13,14]. HSCs are the main fibrogenic cells of the liver and, upon activation, acquire a myofibroblast-like phenotype that drives ECM remodelling [15]. This process involves key ECM components and remodelling enzymes, including collagen type I alpha 1 chain (COL1A1), collagen type I alpha 2 chainCOL1A1, (COL1A2), fibronectin 1 (FN1), and matrix metalloproteinases (MMPs) [16,17,18]. sEVs may also promote EMT-related plasticity by modulating adhesion molecules (E-cadherin (E-CAD), N-cadherin (N-CAD)) and cytoskeletal markers such as vimentin (VIM), thereby enhancing migratory phenotypes [16,17]. Hepatocytes also contribute to metastatic progression by undergoing phenotypic reprogramming in response to tumour-derived sEVs, associated with EMT-related factors such as Twist family BHLH transcription factor 1 (TWIST1) and vesicle-associated proteins, including flotillin 1 (FLOT1) [18,19].

Building on this concept, EMT is increasingly appreciated as a highly plastic process in which cells undergoing partial EMT retain key epithelial features while simultaneously acquiring mesenchymal traits [20]. These intermediate states are associated with increased metastatic potential, collective migration, and therapeutic resistance, and are characterized by non-canonical regulation of classical epithelial and mesenchymal markers [21]. However, despite these advances, how PDAC-derived sEVs coordinate fibrogenesis and EMT-related plasticity within the hepatic microenvironment before metastatic colonisation remains poorly defined [22,23]. Whether distinct hepatic cell populations differentially respond to tumour-derived sEVs, and how these responses contribute to pre-metastatic niche formation, remains unclear.

The present study aimed to define the impact of PDAC-derived sEVs, including those isolated from patient serum, on fibrotic remodelling and EMT-related plasticity in hepatic cells, focusing on hepatocytes (HEPA-RG) and hepatic stellate cells (LX-2). By comparing these models, we sought to identify cell-type-specific responses and delineate mechanisms of hepatic reprogramming within the liver pre-metastatic microenvironment.

## 2. Results

### 2.1. Characterisation and Functional Effects of PDAC-Derived sEVs in Hepatic Cell Models

sEVs derived from MIA-PaCa2 and PANC-1 cell lines displayed the expected physicochemical features consistent with sEVs (Figure S1A–F). TEM analysis confirmed a typical round, cup-shaped morphology with diameters < 200 nm. DLS measurements showed mean hydrodynamic diameters of 112 ± 11 nm and 130 ± 15 nm for MIA-PaCa2 and PANC-1 sEVs, respectively. Both vesicle populations exhibited a negative ζ-potential, indicating good colloidal stability. Western blot analysis confirmed the presence of canonical sEVs markers (HSP70, CD63, CD9) and the absence of the endoplasmic reticulum marker Calnexin, supporting the enrichment of sEVs in both preparations.

Quantitative analysis showed comparable sEVs yields between the two cell lines. sEVs isolated from 40 mL of conditioned medium displayed similar EV-associated protein content, with mean values of 1.10 ± 0.21 µg for MIA-PaCa2 and 1.14 ± 0.15 µg for PANC-1. Consistently, particle concentrations were also comparable, with (5.64 ± 0.42) × 10^7^ particles/mL and (5.78 ± 0.36) × 10^7^ particles/mL for MIA-PaCa2 and PANC-1 sEVs, respectively.

**Figure 1:**
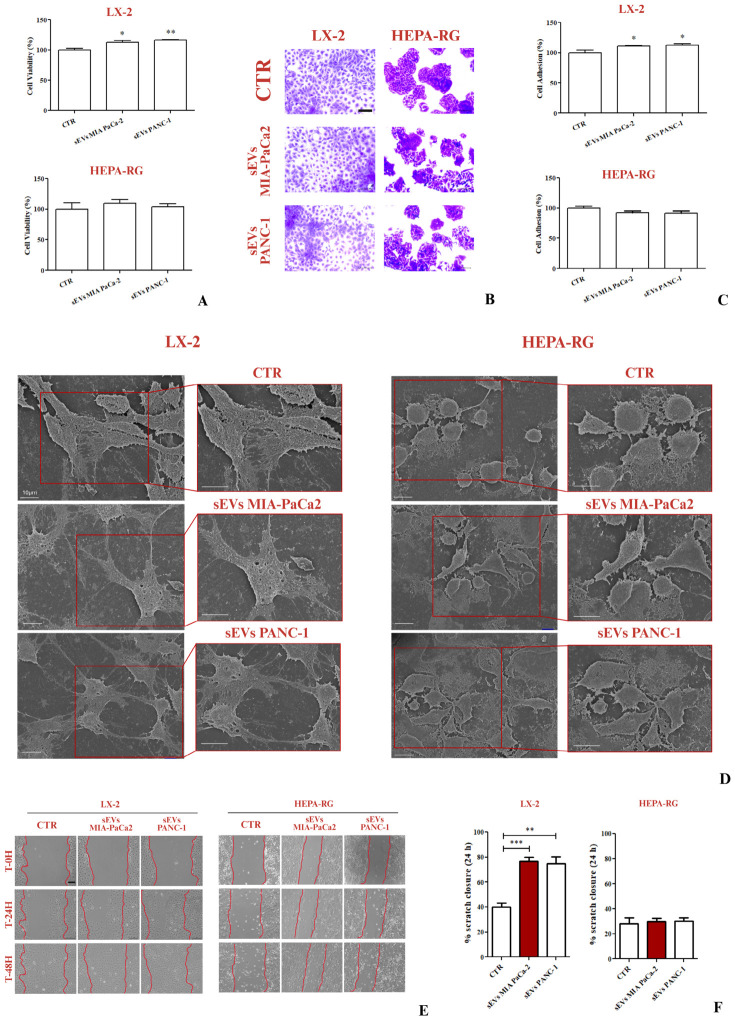
Effects of PDAC-derived sEVs on liver cell viability, morphology, and migration. (**A**) MTS assay of LX-2 and HEPA-RG cells after 72 h sEVs treatment. (**B**) Representative Crystal Violet staining. (**C**) Quantification of cell adhesion (%). (**D**) SEM images showing sEVs-induced morphological changes (scale bar: 10 µm). (**E**) Scratch assay images at 0, 24, and 48 h (scale bar: 200 µm). (**F**) Quantification of wound closure at 24 h. Data are mean ± SD (≥3 experiments). * *p* < 0.05, ** *p* < 0.01, *** *p* < 0.001 vs. control.

LX-2 and HEPA-RG cells were treated for 72 h, and cellular metabolic activity was assessed by MTS assay (Figure 1A). LX-2 cells showed a significant increase in viability following treatment with tumour-derived sEVs compared to control (CTR). Specifically, MIA-PaCa2-derived sEVs significantly increased cell viability (*p* < 0.05), as did PANC-1-derived sEVs (*p* < 0.01). In contrast, no significant changes were observed in HEPA-RG cells.

Crystal Violet staining showed a similar increase in adherent cell biomass (Figure 1B,C). LX-2 cells exhibited a significant increase in adherent cell density following treatment with both MIA-PaCa2- and PANC-1-derived sEVs (*p* < 0.05), whereas HEPA-RG cells did not show significant differences.

The morphological changes induced by tumour-derived sEVs were further evaluated by SEM (Figure 1D), allowing visualisation of the three-dimensional organisation of the cell monolayer. Under control conditions, LX-2 cells exhibited a typical spindle-shaped fibroblastic morphology. Following treatment, LX-2 cells showed evident morphological alterations, losing their elongated shape and acquiring a more flattened, epithelioid appearance.

Similarly, HEPA-RG cells, which display a cuboidal epithelial morphology, showed marked changes after sEVs exposure, appearing more elongated and irregular compared to CTR.

The effects of PDAC-derived sEVs on cell migration were assessed by scratch assay (Figure 1E). In LX-2 cells, sEVs treatment significantly accelerated wound closure at 24 h and 48 h compared to CTR (*p* < 0.001 and *p* < 0.01, respectively), indicating enhanced migratory capacity, as confirmed by quantitative analysis (Figure 1F). In contrast, HEPA-RG cells did not show significant changes in wound closure, with migration rates comparable to CTR (Figure 1F).

### 2.2. Modulation of ECM and EMT Markers by PDAC Cell Line-Derived sEVs in Hepatic Cell Models

Based on the morphological changes observed after treatment with PDAC-derived sEVs, the transcriptional modulation of genes involved in ECM remodelling and EMT was evaluated in hepatic cell models.

In LX-2 cells, RT-qPCR analysis showed modulation of ECM-related genes, with MIA-PaCa2-derived sEVs upregulating MMP9 (*p* < 0.05) and PANC-1-derived sEVs downregulating COL1A1, COL1A2, and FN1 (*p* < 0.05, *p* < 0.01, and *p* < 0.01, respectively) (Figure S2A). Both treatments significantly reduced VIM and ACTA2 expression (*p* < 0.05 and *p* < 0.001), whereas CDH1 and CDH2 levels were not significantly changed (Figure S2B,C). In HEPA-RG cells, sEVs induced a significant reduction in VIM (*p* < 0.01) and an increase in ACTA2 (*p* < 0.001), reaching significance with PANC-1-derived sEVs, whereas no changes were observed for CDH1, CDH2, TWIST1, and FLOT1 (Figure S2D,E).

**Figure 2:**
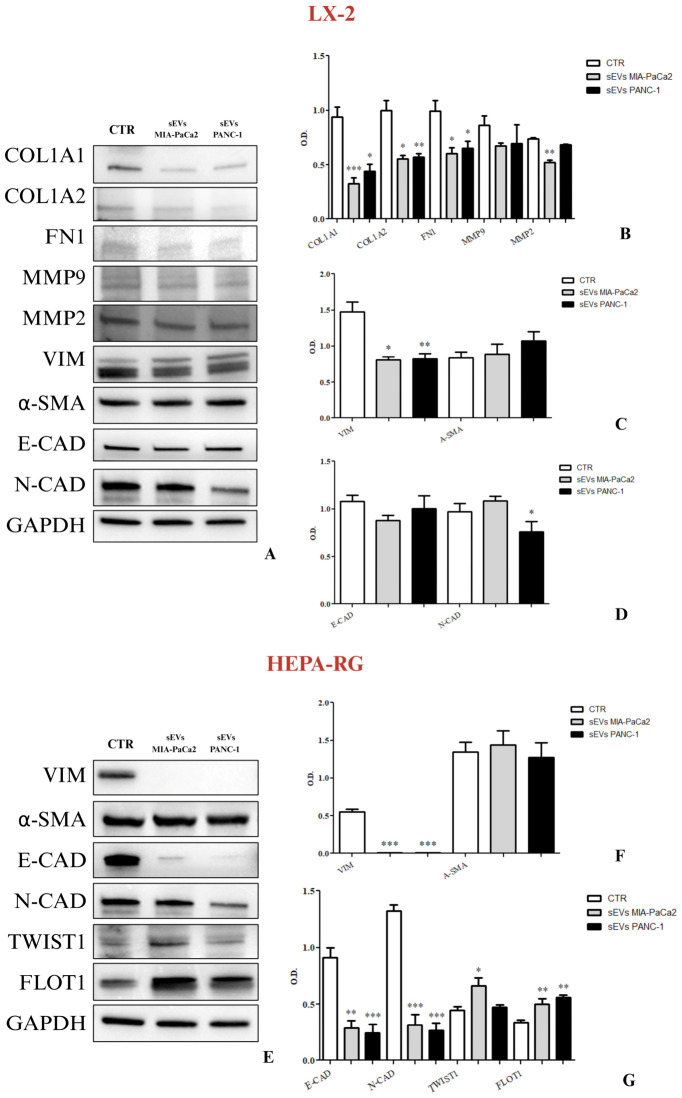
Effects of PDAC cell line-derived sEVs on ECM remodelling and EMT marker protein expression in hepatic cell models. (**A**–**D**) Western blot analysis in LX-2 cells showing ECM proteins and EMT markers, with densitometric quantification. (**E**–**G**) Western blot analysis in HEPA-RG cells showing EMT-related proteins, TWIST1, and FLOT1, with quantification. Protein levels are expressed relative to control. Data are mean ± SD (≥3 experiments). * *p* < 0.05, ** *p* < 0.01, *** *p* < 0.001 vs. control.

Consistent with these findings, Western blot analysis confirmed modulation of ECM- and EMT-related proteins (Figure 2).

In LX-2 cells, exposure to sEVs derived from PDAC cell lines resulted in a significant reduction in fibrogenic ECM proteins. COL1A1 levels decreased following treatment with MIA-PaCa2-derived (*p* < 0.001) and PANC-1-derived sEVs (*p* < 0.05). A similar trend was observed for COL1A2 and FN1 compared to CTR (*p* < 0.05, *p* < 0.01) (Figure 2A,B). Analysis of cytoskeletal and activation markers showed that α-SMA levels remained unchanged, whereas VIM expression was significantly reduced following exposure to both sEVs (*p* < 0.05, *p* < 0.01) (Figure 2A–C). In addition, PANC-1-derived sEVs induced a significant decrease in the adhesion marker N-CAD (*p* < 0.05) (Figure 2A–D).

In HEPA-RG cells, Western blot analysis confirmed a marked reduction in VIM levels after treatment with both PDAC-derived sEVs (*p* < 0.001), while α-SMA remained unchanged (Figure 2E,F). Both treatments also reduced E-CAD and N-CAD expression compared to CTR (Figure 2E–G). In contrast, the level of the EMT-associated protein FLOT1 was significantly increased (*p* < 0.01), while TWIST1 was increased only following treatment with MIA-PaCa2-derived sEVs (*p* < 0.05).

### 2.3. Characterisation and Functional Effects of Patient-Derived sEVs in Hepatic Cell Models

To determine whether the observations obtained with PDAC cell line-derived sEVs could be recapitulated in clinically relevant samples, a cohort of 25 patients (*n* = 25) with histologically confirmed PDAC was included in the study. The cohort comprised 13 females (52%) and 12 males (48%), with a mean age at diagnosis of 65.0 ± 11.1 years. Detailed clinicopathological characteristics of the PDAC patient cohort are reported in Supplementary Table S3. Due to the limited sample size and the heterogeneity of the available clinicopathological characteristics, patient-derived serum sEVs were pooled for subsequent functional and molecular analyses. This strategy was used to reduce the impact of inter-individual variability and to avoid overinterpretation of underpowered patient-specific differences. Therefore, the observed effects should be interpreted as the overall biological activity of circulating sEVs from PDAC patients rather than as patient-specific responses.

**Figure 3:**
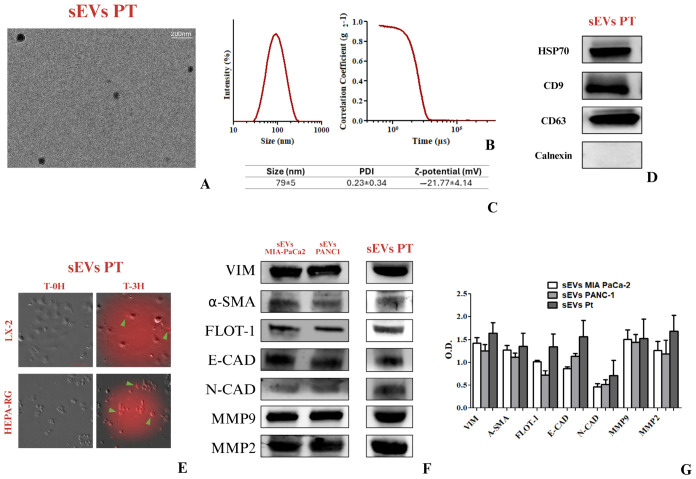
Characterisation of serum-derived sEVs (PT) and protein cargo analysis. (**A**) TEM images of serum-derived sEVs (scale bar: 200 nm). (**B**) Size distribution by DLS. (**C**) Summary of particle size, PDI, and ζ-potential. (**D**) Western blot of sEVs markers (HSP70, CD9, CD63) and negative control (Calnexin). (**E**) Uptake of labelled sEVs by LX-2 and HEPA-RG cells at 0 and 3 h (scale bar: 50 µm). Green arrowheads indicate fluorescently labeled internalized sEVs. (**F**,**G**) Western blot and quantification of EMT- and ECM-related proteins in PDAC cells and derived sEVs. Data are mean ± SD (≥3 experiments).

sEVs isolated from PDAC patient serum displayed the expected physicochemical characteristics of sEVs (Figure 3A–D). TEM analyses confirmed a typical round, cup-shaped morphology, while DLS measurements showed a homogeneous size distribution with a mean diameter of 79 ± 5 nm and a PDI of 0.23 ± 0.04. Vesicle populations exhibited a negative ζ-potential, indicating good colloidal stability. The presence of canonical sEVs markers and the absence of Calnexin further confirmed successful enrichment of sEVs. Quantitative analysis showed that sEVs derived from patient serum had higher protein content (4.53 ± 1.75 µg from 500 µL serum) and particle concentration ((1.87 ± 2.11) × 10^11^ particles/mL) compared to cell line-derived sEVs.

LX-2 and HEPA-RG cells were incubated with fluorescently labelled patient-derived sEVs to evaluate cellular uptake. Vesicle internalisation was evident as early as 3 h, as indicated by intracellular fluorescent signals (Figure 3E), supporting the rapid uptake of circulating PDAC-derived sEVs.

Western blot analysis showed the presence of ECM- and EMT-related proteins in both PDAC cell lines and their corresponding sEVs, including those isolated from patient serum (Figure 3F,G). VIM, α-SMA, FLOT1, E-CAD, N-CAD, MMP-9, and MMP-2 were detected across all samples. Densitometric analysis revealed comparable expression patterns, with no significant differences between cell line-derived and patient-derived sEVs, suggesting a similar protein cargo composition.

The metabolic effects of PDAC-derived sEVs were evaluated by cell viability assays in HEPA-RG and LX-2 cells treated with increasing doses (2.5 × 10^3^, 5 × 10^3^, and 1 × 10^4^ sEVs per cell) over time (24–96 h).

**Figure 4:**
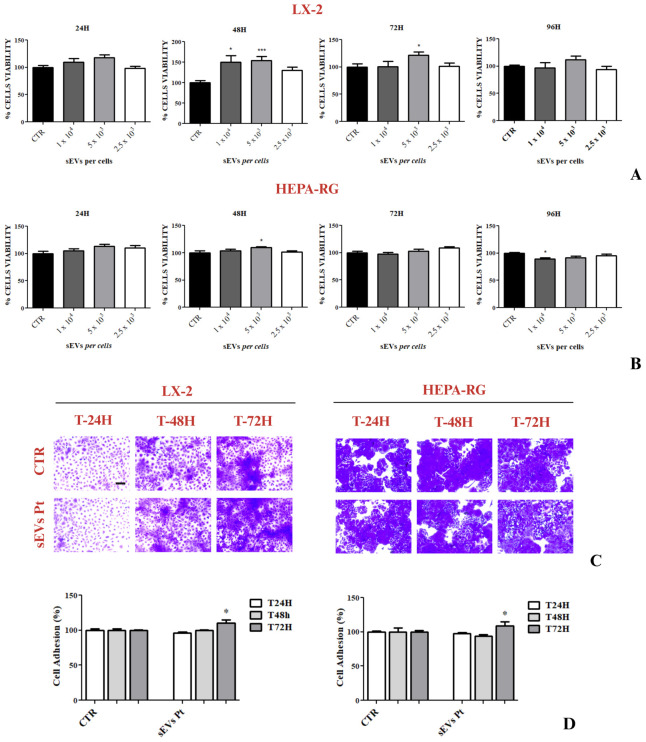
Effect of PDAC-derived sEVs on cell viability in HEPA-RG and LX-2 cells. (**A**,**B**) MTS assay of LX-2 and HEPA-RG cells treated with increasing sEVs doses over time. (**C**) Crystal Violet staining showing changes in cell adhesion (scale bar: 100 µm). (**D**) Quantification of cell adhesion (%). White, light gray, and dark gray bars indicate 24 h, 48 h, and 72 h, respectively. Data are mean ± SD (≥3 experiments). * *p* < 0.05, *** *p* < 0.001 vs. control.

In LX-2 cells, sEVs exposure elicited a more pronounced response compared to HEPA-RG cells. At 48 h, treatment with 5 × 10^3^ and 1 × 10^4^ sEVs per cell significantly increased cell viability (*p* < 0.05 and *p* < 0.001, respectively), indicating enhanced metabolic activity. A similar increase was observed at 72 h at the intermediate dose (*p* < 0.05), whereas no significant differences were detected at 24 h or 96 h compared to CTR (Figure 4A).

In HEPA-RG cells, sEVs treatment did not induce cytotoxic effects at any tested concentration or time point. Cell viability remained comparable to control at 24 and 72, while a modest but significant increase was observed at 48 h at the intermediate dose (*p* < 0.05), suggesting a mild stimulatory effect on metabolic activity. A slight reduction in viability was detected at 96 h at the highest dose (*p* < 0.05), although values remained close to CTR levels (Figure 4B).

These findings are consistent with those observed using PDAC cell line-derived sEVs (Figure 1A), indicating comparable effects on cellular metabolic activity in hepatic cell models. Based on these dose–response results, the intermediate concentration (5 × 10^3^ sEVs per cell) was selected for subsequent experiments, as it provided a balance between biological activity and absence of cytotoxicity.

Consistent with the MTS results, Crystal Violet staining showed a time-dependent increase in adherent cell biomass following treatment with PDAC patient-derived sEVs (5 × 10^3^ sEVs per cell). In LX-2 cells, no significant differences were observed at 24 h or 48 h, whereas a moderate but significant increase in adhesion was detected at 72 h (*p* < 0.05) (Figure 4D). A similar trend was observed in HEPA-RG cells, with adhesion comparable to CTR at earlier time points and a significant increase at 72 h (*p* < 0.05) (Figure 4D).

**Figure 5:**
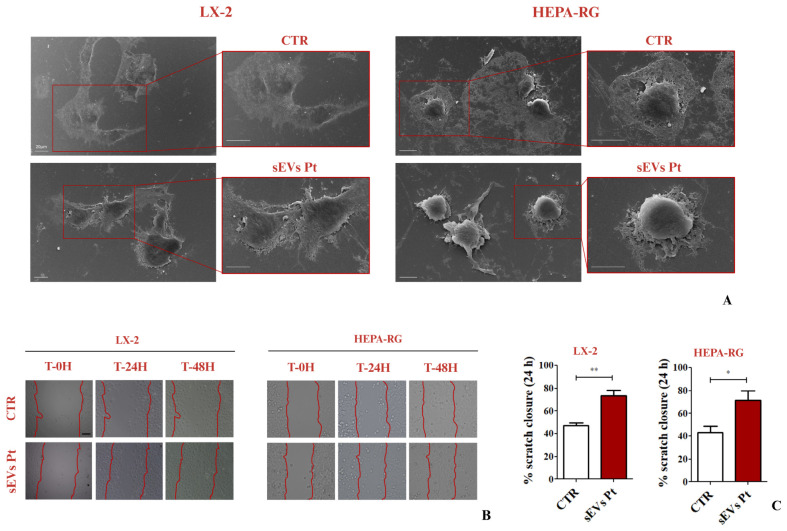
Effects of PDAC patient-derived sEVs on cell morphology and migration in LX-2 and HEPA-RG cells. (**A**) SEM images of LX-2 and HEPA-RG cells showing sEVs-induced morphological changes (scale bar: 20 µm). (**B**) Scratch assay images at 0, 24, and 48 h (scale bar: 200 µm). (**C**) Quantification of wound closure at 24 h. Data are mean ± SD (≥3 experiments). * *p* < 0.05, ** *p* < 0.01 vs. control.

SEM analysis (Figure 5A) showed that, under CTR conditions, LX-2 cells exhibited a typical elongated morphology with a well-organised monolayer. Following treatment, cells appeared more flattened and irregular, with reduced cell–cell contacts. Similarly, HEPA-RG cells displayed a well-spread morphology under CTR conditions, whereas exposure to sEVs induced a more elongated, irregular phenotype with reduced adhesion.

Cell migration was assessed using a wound-healing assay (Figure 5B). In LX-2 cells, sEVs treatment significantly accelerated wound closure, particularly at 24 h, as confirmed by quantitative analysis (*p* < 0.01). A similar pro-migratory effect was observed in HEPA-RG cells, with increased wound closure at 24 h (*p* < 0.05) (Figure 5C).

### 2.4. Modulation of ECM and EMT Markers by PDAC Patient-Derived sEVs in Hepatic Cell Models

Following the same experimental approach, the transcriptional response induced by patient-derived sEVs was evaluated in hepatic cell models.

In LX-2 cells, no significant modulation of ECM-related genes (COL1A1, COL1A2, FN1) and matrix remodelling enzymes (MMP9, MMP2) was observed (Figure S3A). However, VIM expression was significantly reduced (*p* < 0.01), while ACTA2 remained unchanged (Figure S3B). Among adhesion markers, CDH1 (E-CAD) was unaffected, whereas CDH2 (N-CAD) was significantly downregulated (*p* < 0.05) (Figure S3C). In HEPA-RG cells, a distinct response pattern was observed (Figure S3D,E). ACTA2 (α-SMA) was significantly upregulated (*p* < 0.05), while no significant changes were detected for VIM or EMT-related genes (CDH1, CDH2, TWIST1, FLOT1).

Western blot and densitometric analyses further validated the modulation of ECM- and EMT-related proteins.

**Figure 6:**
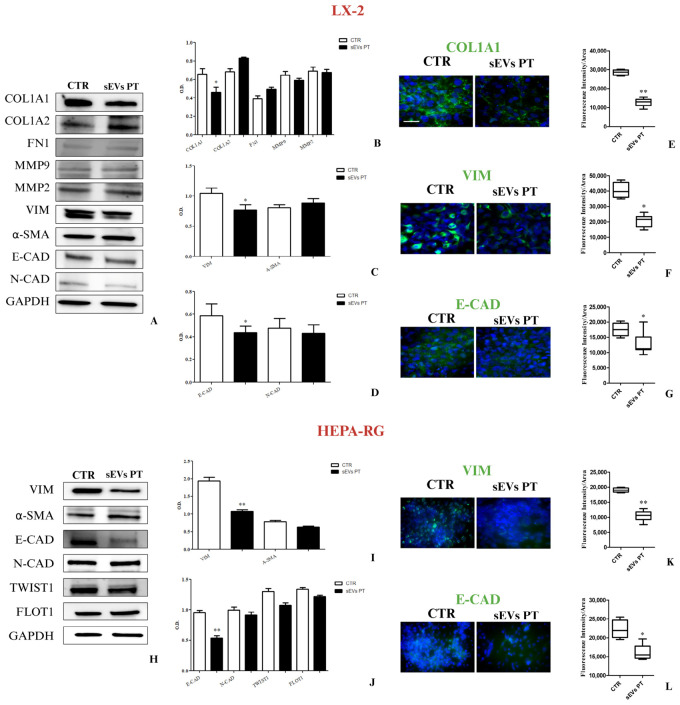
Effects of serum PDAC patients-derived sEVs on ECM remodelling and EMT marker protein in hepatic cell models. (**A**–**D**) Western blot and densitometric analysis of ECM proteins and EMT markers in LX-2 cells. (**E**–**G**) Immunofluorescence and quantification of COL1A1, VIM, and E-CAD (scale bar: 20 µm). (**H**–**J**) Western blot of EMT-related proteins, TWIST1, and FLOT1 in HEPA-RG cells. (**K**,**L**) Immunofluorescence and quantification of VIM and E-CAD (scale bar: 20 µm). Data are mean ± SD (≥3 experiments). * *p* < 0.05, ** *p* < 0.01 vs. control.

In LX-2 cells, COL1A1 levels were significantly reduced compared to CTR (*p* < 0.05), whereas COL1A2, FN1, MMP2, and MMP9 did not show significant changes (Figure 6A,B). Analysis of cytoskeletal and activation markers showed a significant reduction in VIM expression (*p* < 0.05), while α-SMA levels remained unchanged (Figure 6A–C). This reduction is consistent with the trend observed with cell line-derived sEVs, whereas α-SMA did not show the coordinated modulation previously detected. A similar trend was observed in HEPA-RG cells, where VIM expression was also significantly (Figure 6H,I), although to a lesser extent than in response to PDAC cell line-derived sEVs (Figure 2).

E-CAD expression was significantly reduced in both LX-2 and HEPA-RG cells (*p* < 0.05 and *p* < 0.01, respectively), whereas N-CAD levels remained comparable to CTR (Figure 6A–D). In HEPA-RG cells, no significant changes were detected for the EMT-associated protein TWIST1 and for the membrane-associated protein FLOT1 following treatment with patient-derived sEVs (Figure 6H–J).

These findings were further supported by immunofluorescence analysis. In LX-2 cells, COL1A1, VIM, and E-CAD fluorescence intensity were significantly reduced in patients’ sEVs-treated cells compared to CTR (*p* < 0.01, *p* < 0.05, and *p* < 0.05, respectively), in agreement with Western blot results (Figure 6E–G). Similarly, in HEPA-RG cells, VIM and E-CAD fluorescence intensity were reduced (*p* < 0.01 and *p* < 0.05, respectively), confirming the modulation of EMT-related markers at the protein level (Figure 6K,L).

**Figure 7:**
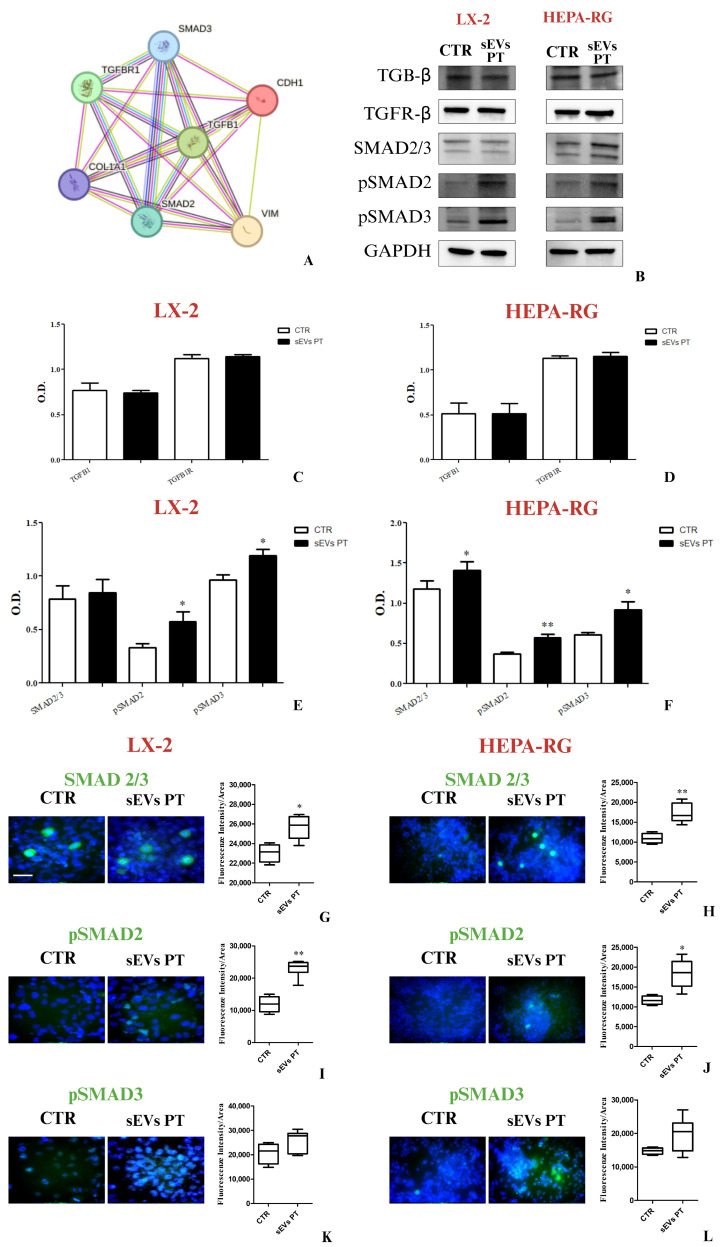
Involvement of the TGFβ1/SMAD signalling pathway in hepatic cell models treated with PDAC patients-derived sEVs. (**A**) STRING network of TGFβ1 signalling and EMT-related proteins. (**B**) Western blot of TGFβ1 pathway components in LX-2 and HEPA-RG cells. (**C**–**F**) Densitometric analysis of TGFβ1/TGFβR1 and SMAD2/3 activation. (**G**–**L**) Immunofluorescence and quantification of SMAD2/3, pSMAD2, and pSMAD3 (scale bar: 20 µm). Data are mean ± SD (≥3 experiments). * *p* < 0.05, ** *p* < 0.01 vs. control.

To elucidate the signalling mechanisms potentially underlying the observed phenotypic changes—although this was not the primary objective of the present study—the activation of the TGF-β/SMAD pathway, a key regulator commonly implicated in the initiation of EMT, was assessed in liver cells following exposure to PDAC-derived sEVs. Protein–protein interaction analysis using STRING (Figure 7A) revealed a highly interconnected network involving TGF-β signalling components (TGFB1, TGFBR1, SMAD2, SMAD3), together with EMT- and ECM-related markers (VIM, CDH1, COL1A1).

Western blot analysis showed that PDAC-derived sEVs did not significantly affect TGF-β1 or TGFβR1 expression in either LX-2 or HEPA-RG cells (Figure 7B–D). Total SMAD2/3 levels remained unchanged in LX-2 cells but were significantly increased in HEPA-RG cells (*p* < 0.05). In contrast, a marked increase in phosphorylated SMAD2 and SMAD3 was observed in both cell lines, with significant upregulation of p-SMAD2 and p-SMAD3 (Figure 7E,F).

These observations were corroborated by immunofluorescence analysis. In LX-2 cells, SMAD2/3 and pSMAD2 fluorescence intensity significantly increased following patients’ sEVs treatment (*p* < 0.05 and *p* < 0.01, respectively), while pSMAD3 levels remained unchanged. A similar pattern was observed in HEPA-RG cells, with increased SMAD2/3 and pSMAD2 (*p* < 0.01 and *p* < 0.05, respectively), but no significant variation in pSMAD3 (Figure 7G–L). Notably, pSMAD2 and pSMAD3 signals showed predominant nuclear localisation upon sEVs exposure, consistent with SMAD pathway modulation.

## 3. Discussion

In the liver, sEVs have been implicated in inflammatory and fibrotic processes associated with metastatic progression [24]. However, their role in coordinating fibrogenesis and EMT-related plasticity within the hepatic microenvironment remains incompletely understood.

This study demonstrates that sEVs from PDAC cell lines or patient samples are rapidly internalised by hepatic cells, inducing extensive, cell type-specific morphological, functional, and molecular reprogramming, consistent with prior reports [25,26], that reflects selective hepatic remodelling rather than a uniform response.

Notably, Costa-Silva et al. demonstrated that PDAC-derived sEVs contribute to the formation of a hepatic pre-metastatic niche by activating Kupffer cells, which secrete TGFβ1 and subsequently promote hepatic stellate cell activation, fibronectin deposition, and ECM remodelling. This cascade facilitates the recruitment of bone marrow-derived cells, ultimately supporting metastatic colonisation [23]. In this context, our findings provide phenotypic evidence of hepatic cell reprogramming compatible with early pre-metastatic niche-associated processes, suggesting that this response may not follow a uniform fibrogenic trajectory but rather unfold as a non-linear and context-dependent process. While hepatic stellate cells are widely recognised as central stromal mediators in tumour progression and metastasis, increasing evidence also points to a significant contribution of the parenchymal compartment [27,28]. Hepatocytes are not passive bystanders but actively and critically regulate the liver microenvironment, responding to tumour-derived signals through pronounced functional adaptation and phenotypic reprogramming [29]. Together, these observations suggest that stromal and parenchymal compartments mount coordinated but distinct responses, potentially contributing to a permissive hepatic microenvironment even in the absence of a full mesenchymal transition. The inclusion of both LX-2 and HEPA-RG cells was intended to distinguish stromal/fibrogenic responses from hepatocyte-like parenchymal adaptations to PDAC-derived sEVs. While LX-2 cells provide a model of stellate cell activation and ECM remodelling, HEPA-RG cells allow assessment of whether the parenchymal compartment may also undergo phenotypic and EMT/plasticity-related changes in response to tumour-derived vesicles. In LX-2 cells, exposure to PDAC-derived sEVs increased metabolic activity, enhanced cell spreading and adhesion, and promoted morphological changes and migration, supporting a dynamically activated stromal phenotype. However, these features do not reflect a classical EMT program, underscoring the need for caution when applying the EMT framework to hepatic stellate cells. Rather, our findings demonstrate that the modulation of markers such as VIM, ACTA2, COL1A1, COL1A2, and FN1 aligns more closely with dynamic changes in activation state and extracellular matrix remodelling capacity than with a canonical epithelial-to-mesenchymal transition process [30,31]. sEVs derived from PDAC cell lines, particularly PANC-1, induced downregulation of COL1A1, COL1A2, and FN1, while α-SMA remained unchanged, suggesting a non-canonical pattern of ECM remodelling. In contrast, patient-derived sEVs exerted a milder and less coordinated effect, mainly reflected by COL1A1 downregulation. Importantly, the lack of significant changes in MMP2 and MMP9 further indicates that this remodelling is not driven by proteolytic activity but rather reflects an early and selective phase of extracellular microenvironment reorganisation. This is consistent with pre-metastatic niche models in PDAC, in which early stromal activation has been proposed to precede matrix degradation [32].

In HEPA-RG cells, PDAC-derived sEVs elicited a distinct response, with minimal effects on metabolic activity but evident morphological changes, increased migration, and selective modulation of adhesion and plasticity-related markers, indicating a predominantly plasticity-driven response. Specifically, sEVs reduced E-CAD and N-CAD levels while increasing TWIST1 and FLOT1, with patient-derived vesicles inducing a similar but less pronounced effect. Together, these findings highlight distinct yet functionally convergent responses across hepatic cell compartments. Consistently, E-CAD downregulation, a key determinant of epithelial integrity, supports the acquisition of a pro-migratory phenotype even in the absence of a full EMT [33].

VIM expression was also reduced, a seemingly paradoxical finding that aligns with emerging evidence of context-dependent and non-canonical EMT-related plasticity [34]. VIM expression is not always linearly associated with tumour aggressiveness but rather exhibits marked heterogeneity and a context-dependent prognostic relevance [35]. Moreover, reduced VIM levels may influence cytoskeletal organisation, cell stiffness, deformability, and adhesion dynamics, thereby potentially facilitating migration in specific cellular contexts, including partial or hybrid EMT-like states [36].

These observations support a cautious interpretation in terms of EMT-like plasticity rather than a fully mesenchymal transition. This is consistent with the current view of EMT as a continuum of intermediate states, in which cells acquire migratory and adaptive features without completing a full mesenchymal program [37,38]. Similar hybrid states have been described in response to tumour-derived sEVs, highlighting their ability to induce context-dependent cellular reprogramming [39].

Patient-derived sEVs, although less potent than those from cell lines, induced qualitatively similar effects, including increased migration and selective modulation of VIM and E-CAD. This reduced magnitude likely reflects the greater heterogeneity of circulating vesicles in vivo [40]. Crucially, this convergence supports the clinical relevance of these findings and argues against an in vitro artifact.

Finally, the results of this study suggest a possible activation of the TGF-β/SMAD pathway, a key regulator of stromal remodelling and EMT-related plasticity [41]. The increase in p-SMAD2/3 levels observed in treated cells indicates the potential involvement of this signalling axis in sEVs-induced reprogramming. However, these observations should be interpreted with caution, as no changes were detected in TGF-β1 or its receptor, while SMAD phosphorylation was selectively modulated, pointing to a non-canonical activation mechanism. Moreover, SMAD signalling may occur independently of TGF-β1, including ligand-independent phosphorylation, with SMAD2 and SMAD3 exerting distinct regulatory roles [42,43].

Tumour-derived and biomimetic vesicles may also represent promising therapeutic platforms due to their biocompatibility, membrane properties, and ability to transport bioactive molecules. In this context, recent vesicle-based strategies have shown potential for targeted drug delivery, chemo-immunotherapy, and phototherapy. [44,45] suggesting that engineered vesicle systems may be exploited to counteract tumour progression.

This study is limited to using in vitro models, which do not fully recapitulate the complexity of the hepatic microenvironment, and by the relatively small and heterogeneous patient cohort. In addition, serum-derived sEVs represent a mixed population of circulating vesicles, not exclusively tumour-derived, which may influence the observed effects [46]. Accordingly, the biological effects observed with patient-derived serum sEVs should be interpreted as the net effect of circulating vesicles from PDAC patients, rather than as effects exclusively attributable to pancreatic tumour-derived vesicles. Although this pooling strategy reduced inter-individual variability, it limited patient-specific interpretation of the observed biological effects. Despite these limitations, our findings provide a basis for further investigation into the mechanisms underlying hepatic microenvironment remodelling. Although selected ECM- and EMT/plasticity-related proteins were assessed in sEVs preparations, the present study did not include comprehensive proteomic, transcriptomic, or miRNA cargo profiling. Comprehensive characterisation of sEVs cargo and validation in more complex multicellular and in vivo models will be essential to better define their role in liver-specific reprogramming and niche formation. In parallel, clinical stratification of patient-derived sEVs may help identify associations with hepatic involvement, metastatic risk, and early liver niche priming, supporting their potential as circulating biomarkers and targets for modulating tumour–liver crosstalk.

## 4. Materials and Methods

### 4.1. Cell Culture

Human hepatic stellate cells (LX-2, RRID: CVCL_5792) and hepatocyte-like cells (HEPA-RG, RRID: CVCL_9720) were cultured under standard conditions. LX-2 cells were maintained in DMEM supplemented with 10% exosome-depleted foetal bovine serum and standard additives. HEPA-RG cells were cultured in hepatocyte-specific medium (Lonza) supplemented with 10% exosome-depleted foetal bovine serum. PDAC cell lines (MIA-PaCa2, RRID: CVCL_0428; PANC-1, RRID: CVCL_0480) were maintained in DMEM supplemented with 10% exosome-depleted foetal bovine serum and standard additives. All cell cultures were maintained at 37 °C in a humidified atmosphere with 5% CO_2_.

### 4.2. Patients

A total of 25 treatment-naïve patients with histologically confirmed PDAC were enrolled at the IRCCS “Saverio de Bellis” Research Hospital (Italy). Tumour staging was defined according to the TNM (Tumour–Node–Metastasis) classification.

The study was conducted in accordance with the Declaration of Helsinki and approved by the local Ethics Committee (protocol no. 1943/CEL-Studio EMT-PDCA). Written informed consent was obtained from all participants. Patients with recurrent disease, prior malignancies, or previous oncological treatments were excluded. Serum samples were collected for subsequent analyses.

### 4.3. sEVs Isolation, Characterisation, and Uptake Assay

sEVs were isolated from conditioned media of MIA-PaCa2 and PANC-1 cells or from patient serum by differential ultracentrifugation, as previously described [24]. For patient-derived samples, sEVs were first isolated and characterized individually in terms of protein content and particle concentration. The individual patient-derived sEVs preparations were then combined into a pooled PDAC patient-derived circulating sEVs preparation, which was used for subsequent functional and molecular assays. Briefly, cell culture supernatants were sequentially centrifuged to remove debris and subsequently ultracentrifuged at 100,000× *g* to pellet sEVs. Vesicles were resuspended in ultrapure water and characterized according to standard criteria, including size distribution and ζ-potential by dynamic light scattering (DLS, Zetasizer Ultra, Malvern Panalytical, Worcestershire, UK), and morphology by transmission electron microscopy (TEM, Jeol JEM-1011 microscope, JEOL USA, Inc., Peabody, MA, USA). Particle concentration was estimated based on light scattering analysis. Samples were stored for subsequent protein analyses.

Cellular uptake of patient-derived sEVs was assessed using a membrane fluorescent labelling kit (ExoGlow™, System Biosciences, Newark, CA, USA), following the manufacturer’s instructions. Labelled sEVs were added to HEPA-RG and LX-2 cells after adhesion, and internalisation was monitored over time by fluorescence microscopy (Cell Discoverer 7, Carl Zeiss Microscopy GmbH, Jena, Germany). The presence of intracellular fluorescence was considered indicative of sEVs uptake. All experiments were performed in triplicate.

### 4.4. Cell Viability Assay

Cell viability was assessed using the MTS metabolic assay and Crystal Violet staining. HEPA-RG and LX-2 cells were seeded in 96-well plates (2 × 10^3^ cells/well) and treated in triplicate with sEVs derived from MIA-PaCa2 and PANC-1 cells (10 µg/mL; ~1 × 10^8^ particles/mL) for up to 72 h, with medium renewal every 24 h. Untreated cells served as controls.

For patient-derived sEVs, cells were exposed to increasing concentrations (2.5 × 10^3^, 5 × 10^3^, and 1 × 10^4^ sEVs per cell) and analysed at different time points (24–96 h). Based on dose–response experiments, the intermediate concentration (5 × 10^3^ sEVs per cell) was selected for subsequent assays. Cell viability was assessed by measuring the quantity of formazan product, which is directly proportional to the number of viable cells, through absorbance at 490 nm using a PerkinElmer Victor Plate Reader (Lier, Belgium).

For Crystal Violet staining, cells were fixed with paraformaldehyde, stained with crystal violet solution, and extensively washed to remove excess dye. Images were acquired to assess cell morphology and adherent cell density using a Nikon Eclipse Ti2 confocal microscope (Nikon Corporation, Tokyo, Japan) in bright field at 20× magnification. For quantitative analysis, the bound dye was solubilised, and absorbance was measured at 595 nm.

### 4.5. Scanning Electron Microscopy (SEM) Analysis

LX-2 and HEPA-RG cells were seeded on silicon wafers and treated with sEVs derived from PDAC cell lines for 72 h under the conditions described above. Following treatment, cells were fixed, dehydrated through a graded ethanol series, and processed for SEM. Samples were sputter-coated with gold (208HR High Resolution Sputter Coater, Ted Pella Inc., Redding, CA, USA) and imaged by field emission SEM (Zeiss Sigma FE-SEM, Carl Zeiss Microscopy GmbH, Jena, Germany) investigation. Representative micrographs were obtained from at least three independent experiments.

### 4.6. Cell Migration Analysis

Cell migration was evaluated using a scratch wound-healing assay. HEPA-RG and LX-2 cells were seeded in 12-well plates, and a linear scratch was generated on the cell monolayer. After removal of cellular debris, cells were treated with sEVs derived from MIA-PaCa2, PANC-1, or PDAC patient serum under the conditions described above. Wound closure was monitored by phase-contrast microscopy at 0, 24, and 48 h.

### 4.7. Gene and Protein Expression Analysis

LX-2 and HEPA-RG cells were treated with sEVs derived from MIA-PaCa2, PANC-1, or the serum of patients with PDAC for 48 h for gene expression analysis and 72 h for protein analysis, with a treatment administered every 24 h. Total RNA was extracted using the RNeasy Mini Kit (QIAGEN, Hilden, Germany), and cDNA was synthesised from 2 μg of total RNA. Real-time PCR was performed using SYBR Green chemistry with validated human primers. Relative gene expression was calculated using the 2^−ΔΔCt^ method, normalised to GAPDH, and expressed relative to control samples. Details of the analysed genes are provided in Supplementary Table S1.

For protein analysis, total lysates were prepared using RIPA buffer supplemented with protease and phosphatase inhibitors, and protein concentration was determined by Bradford assay. Equal amounts of protein (25 μg) were separated by SDS-PAGE and transferred onto PVDF membranes. Membranes were incubated with specific primary antibodies followed by HRP-conjugated secondary antibodies, and proteins were detected by chemiluminescence. Densitometric analysis was performed using Image Lab 5.2.1 software, with normalisation to GAPDH. Details of the analysed proteins are provided in Supplementary Table S2.

### 4.8. Immunofluorescence Analysis

LX-2 and HEPA-RG cells were treated for 72 h with sEVs derived from PDAC patient serum under the conditions described above. Following treatment, cells were fixed with paraformaldehyde, permeabilised with Triton X-100 (Sigma, St. Louis, MI, USA), and blocked to prevent non-specific binding.

Immunofluorescence staining was performed using specific primary antibodies against E-CAD, VIM, COL1A1, SMAD2/3, p-SMAD2, and p-SMAD3 in LX-2 cells, and E-CAD, VIM, SMAD2/3SMAD family member 2/3 (SMAD2/3), phosphorylated SMAD2/3 (p-SMAD2/3), p-SMAD2, and p-SMAD3 in HEPA-RG cells. After incubation with appropriate fluorophore-conjugated secondary antibodies, nuclei were counterstained with DAPI. Fluorescence images were acquired using a Cell Discoverer 7 system (Carl Zeiss Microscopy GmbH, Jena, Germany).

### 4.9. Bioinformatic Analysis

Protein–protein interaction analysis was performed using the STRING database (v12.0) to explore functional relationships among selected proteins (SMAD2, SMAD3, TGFB1, TGFB1R, CDH1, VIM, and COL1A1). Interactions were analysed using a medium confidence score (0.4), integrating experimental data, curated databases, and co-expression evidence.

### 4.10. Statistical Analysis

Data are presented as mean ± standard deviation (SD) from at least three independent biological replicates, unless otherwise specified. Statistical analyses were performed using GraphPad Prism (v10.4.1). Normality was assessed using the Shapiro–Wilk test. Comparisons among multiple groups were performed using one-way or two-way ANOVA, as appropriate, followed by Dunnett’s post hoc test. For pairwise comparisons, an unpaired two-tailed Student’s *t*-test was used. A *p*-value < 0.05 was considered statistically significant (* *p* < 0.05, ** *p* < 0.01, *** *p* < 0.001).

## 5. Conclusions

This study compellingly demonstrates that PDAC-derived sEVs orchestrate selective, cell type-specific remodelling of the hepatic microenvironment. These robust findings reveal coordinated yet distinct responses featuring stromal activation with enhanced viability and motility, coupled with parenchymal reprogramming marked by altered adhesion, morphology, and migratory capacity. Collectively, these data establish a dynamic, context-dependent model of hepatic niche priming wherein tumour-derived sEVs decisively sculpt a pro-metastatic liver milieu—manifesting as EMT-like or partial/hybrid plasticity rather than full mesenchymal transition.

## Data Availability

All data are presented in this publication.

## Supplementary Materials

The following supporting information can be downloaded at: https://www.mdpi.com/article/10.3390/ijms27125270/s1.

## Abbreviations

The following abbreviations are used in this manuscript:

PDAC	pancreatic ductal adenocarcinoma
sEVs	small extracellular vesicles; ECM, extracellular matrix
EMT	epithelial–mesenchymal transition
SEM	scanning electron microscopy
VIM	Vimentin
E-CAD	E-cadherin
N-CAD	N-cadherin
TME	tumour microenvironment
EVs	extracellular vesicles
lEVs	large extracellular vesicles
HSCs	hepatic stellate cells
COL1A1	collagen type I alpha 1 chain
COL1A2	collagen type I alpha 2 chain
FN1	fibronectin 1
MMPs	matrix metalloproteinases
TWIST1	Twist family BHLH transcription factor 1
FLOT1	flotillin 1
RRID	Research Resource Identifier
TNM	tumour–node–metastasis
DLS	dynamic light scattering
TEM	transmission electron microscopy
MTS	3-(4,5-dimethylthiazol-2-yl)-5-(3-carboxymethoxyphenyl)-2-(4-sulfophenyl)-2H-tetrazolium
CTR	Control
qRT-PCR	quantitative real-time polymerase chain reaction
ACTA2	actin alpha 2, smooth muscle
α-SMA	alpha-smooth muscle actin
TGF-β	transforming growth factor beta
TGFBR1	transforming growth factor beta receptor 1
SMAD2/3	SMAD family member 2/3
p-SMAD2/3	phosphorylated SMAD2/3
SD	standard deviation
ANOVA	analysis of variance
GAPDH	glyceraldehyde-3-phosphate dehydrogenase
