# Supplementary material for: Pancreatic Cancer-Derived Small Extracellular Vesicles Remodel Hepatic Pre-Metastatic Niche via Hybrid Epithelial–Mesenchymal States
Source: Int J Mol Sci. 2026 Jun 10;27(12):5270. doi: 10.3390/ijms27125270 (PMC13299023; doi:10.3390/ijms27125270)

Article

Pancreatic Cancer-Derived Small Extracellular Vesicles Remodel Hepatic Pre-Metastatic Niche via Hybrid Epithelial–Mesenchymal States

Francesco Balestra [1], Giorgia Panzetta [1], Maria De Luca [1], Federica Rizzi [2–3], Anna Ancona [4], Ilaria Grassi [5], Roberto Comparelli [2–3], Maria Lucia Curri [2–3–6], Gianluigi Giannelli [7], Nicoletta Depalo [2–3†] and Maria Principia Scavo [1†*]

[1] Laboratory of Molecular Medicine, National Institute of Gastroenterology IRCCS S. deBellis, Via Turi 27, Castellana Grotte (Bari); francesco.balestra@irccsdebellis.it (F.B.); giorgia.panzetta@irccsdebellis.it (G.P.); maria.deluca@irccsdebellis.it (M.D.L.); maria.scavo@irccsdebellis.it (M.P.S.).

[2] Institute for Chemical-Physical Processes, Italian National Research Council (IPCF)-CNR SS Bari, Via Orabona, 70125 Bari, Italy; nicoletta.depalo@cnr.it (N.D.); federica.rizzi@cnr.it (F.R.).

[3] National Interuniversity Consortium of Materials Science and Technology (INSTM), Bari Research Unit, Via Orabona 4, 70126 Bari, Italy;

[4] Core Facility Biobank, National Institute of Gastroenterology IRCCS "S. de Bellis", Research Hospital, Via Turi 27, Castellana Grotte, 70013 Bari, Italy; anna.ancona@irccsdebellis.it (A.A.)

[5] Department of Pathology, National Institute of Gastroenterology IRCCS "S. de Bellis", Research Hospital, Via Turi 27, Castellana Grotte, 70013 Bari, Italy; ilaria.grassi@irccsdebellis.it (I.G.)

[6] Department of Chemistry, University of Bari, Via Orabona 4, 70125 Bari, Italy; marialucia.curri@uniba.it (M.L.C).

[7] Scientific Direction, National Institute of Gastroenterology IRCCS S. deBellis, Via Turi 27, Castellana Grotte (Bari); gianluigi.giannelli@irccsdebellis.it (G.G.)

† These authors also contributed equally to this work.

* Correspondence: maria.scavo@irccsdebellis.it; Tel.: +39-0804994691

Table S1. List of genes analysed by RT-PCR, including gene name, assay code, and RefSeq accession numbers. The checkmark symbol (✓) indicates the cell line(s) in which the gene was analysed.

Name	Code	RefSeq	LX-2	HEPA-RG
**CDH1**	qHsaCID0015365	NM_004360;	✓	✓
**CDH2**	qHsaCID0015189	NC_000018.9; NG_011959.1; NT_010966.14;	✓	✓
**VIM**	qHsaCID0012604	NC_000010.10; NG_012413.1; NT_008705.16;	✓	✓
**ACTA2**	qHsaCID0013300	NC_000010.10; NG_011541.1; NT_030059.13;	✓	✓
**FN1**	qHsaCED003611	NC_000002.11; NT_005403.17; NG_012196.1;	✓	
**COL1A1**	qHsaCED0043248	NC_000017.10; NG_007400.1; NT_010783.15;	✓	
**COL1A2**	qHsaCED0003988	NC_000007.13; NG_007405.1; NT_007933.15;	✓	
**MMP2**	qHsaCID0015623	NC_000016.9; NT_010498.15; NG_008989.1;	✓	
**MMP9**	qHsaCID0011597	NC_000020.10; NG_011468.1; NT_011362.10;	✓	
**TWIST1**	qHsaCED0003856	NC_000007.13; NG_008114.1; NT_007819.17;		✓
**FLOT1**	qHsaCED0037092	NC_000006.11; NT_007592.15; NT_113891.2; NT_167244.1; NT_167245.1; NT_167246.1; NT_167247.1; NT_167248.1; NT_167249.1;		✓

Table S2. List of primary antibodies analysed. The table includes antibody name, catalogue code, host species/isotype, working dilution, and the specific application. A checkmark (✓) indicates the cell line or application (sEVs characterization, LX-2, or HEPA-RG).

Name	Code	Host/Isotype	Dilution	EVs characterization	LX-2	HEPA-RG
HSP70 Monoclonal Antibody (3A3)	(Thermo Fisher Scientific Cat# MA3-006, RRID:AB_325454)	Mouse	1:1000	✓		
Anti-CD63 antibody [TS63] - BSA and Azide free	(Abcam Cat# ab59479, RRID:AB_940915)	Mouse	1:500	✓		
Anti-CD9 antibody [EPR23105-121]	(Abcam Cat# ab236630, RRID:AB_2922400)	Rabbit	1:1000	✓		
Calnexin Monoclonal Antibody (3A3)	(Thermo Fisher Scientific Cat# MA3-027, RRID:AB_2069043)	Mouse	1:1000	✓		
E-Cadherin (24E10) Rabbit mAb	(Cell Signaling Technology Cat# 3195, RRID:AB_2291471)	Rabbit	1:500		✓	✓
Anti-N Cadherin antibody - Intercellular Junction Marker	(Abcam Cat# ab18203, RRID:AB_444317)	Rabbit	1:500		✓	✓
Anti-MMP2 antibody	(Thermo Fisher Scientific Cat# 436000, RRID:AB_2532214)	Mouse	1:500		✓	
Anti-MMP9 antibody	(Thermo Fisher Scientific Cat# MA5-15886, RRID:AB_11157246)	Rabbit	1:1000		✓	
Vimentin (D21H3) XP® Rabbit mAb	(Cell Signaling Technology Cat# 5741, RRID:AB_10695459)	Rabbit	1:1000		✓	✓
Anti-α-Smooth Muscle Actin (ACTA2) Antibody	(Sigma-Aldrich Cat# A5228, RRID:AB_262054)	Mouse	1:1000		✓	✓

Fibronectin Polyclonal Antibody	(Thermo Fisher Scientific Cat# PA5-29578, RRID:AB_2547054)	Rabbit	1:500	✓	
COL1A1 (E8I9Z) Rabbit mAb	(Cell Signaling Technology Cat# 91144, RRID:AB_2800169)	Rabbit	1:1000	✓	
Anti-COL1A2 antibody	(Abcam Cat# ab96723, RRID:AB_10679394)	Rabbit	1:500	✓	
TWIST1 Monoclonal Antibody (2F8E7)	(Thermo Fisher Scientific Cat# MA5-17195, RRID:AB_2538666)	Mouse	1:500		✓
Flotillin-1 (D2V7J) XP® Rabbit mAb	(Cell Signaling Technology Cat# 18634, RRID:AB_2773040)	Rabbit	1:1000		✓
Smad 2/3 Antibody	(Cell Signaling Technology Cat#3102, RRID: AB_10698742)	Rabbit	1:1000	✓	✓
Phospho-SMAD2 (Ser465/Ser467) (E8F3R) Rabbit Monoclonal Antibody	(Cell Signaling Technology Cat#18338, RRID: AB_2798798)	Rabbit	1:1000	✓	✓
Phospho-SMAD3 (Ser423/425) (C25A9) Rabbit Monoclonal Antibody	(Cell Signaling Technology Cat#9520, RRID: AB_2193207)	Rabbit	1:1000	✓	✓
TGF beta-1 Monoclonal Antibody (TB21)	(Thermo Fisher Scientific Cat# MA5-18023, RRID: AB_2539407)	Mouse	1:500	✓	✓
Anti-TGF beta Receptor I antibody	(Abcam Cat# ab235178, RRID: AB_2895230)	Rabbit	1:1000	✓	✓

Figure S1. Characterization of sEVs derived from pancreatic cancer cell lines.

(A–B) TEM images of sEVs isolated from MIA-PaCa2 and PANC-1 cell lines (scale bar = 200 nm). Images are representative of three independent isolations. (C–D) Dynamic light scattering (DLS) analysis showing the size distribution of sEVs. (E) Table summarizing the mean particle size, polydispersity index (PDI), and ζ-potential of sEVs. Data are presented as mean ± SD from three independent measurements. (F) Western blot analysis of canonical positive sEV markers (HSP70, CD63, and CD9) and Calnexin as a negative control in sEVs derived from MIA-PaCa2 and PANC-1 cells. Results are representative of at least three independent isolations.

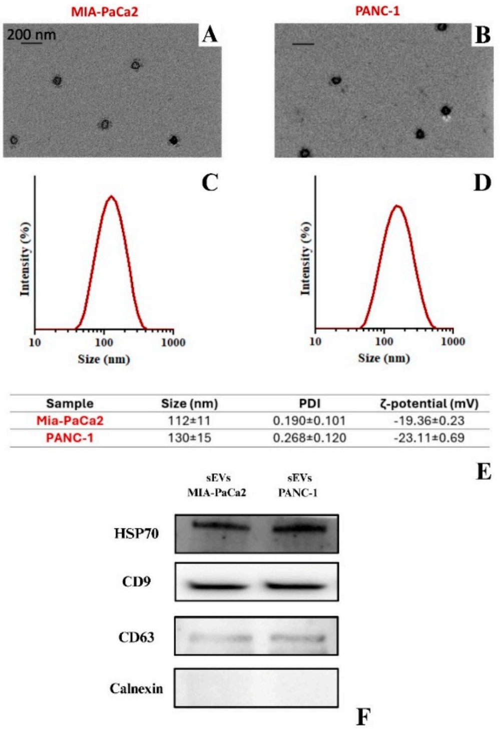

Figure S2. Effects of PDAC cell line-derived sEVs on ECM remodelling and EMT marker expression in hepatic cell models. (A–C) qRT–PCR analysis in LX-2 cells treated with sEVs from MIA-PaCa2 or PANC-1 cells. (A) ECM-related genes (COL1A1, COL1A2, FN1) and remodelling enzymes (MMP9, MMP2). (B) Mesenchymal/activation markers VIM and ACTA2 (α-SMA). (C) Adhesion markers CDH1 (E-cadherin) and CDH2 (N-cadherin). (D–E) qRT–PCR analysis in HEPA-RG cells treated with PDAC-derived sEVs. (D) VIM and ACTA2 expression. (E) CDH1, CDH2, TWIST1 (transcription factor), and FLOT1 (flotillin-1). Expression levels are shown as fold change relative to untreated control (CTR). Data represent mean ± SD from three independent experiments. Statistical significance was determined by one-way ANOVA followed by Dunnett's multiple comparisons test. *p < 0.05, **p < 0.01, ***p < 0.001 vs CTR.

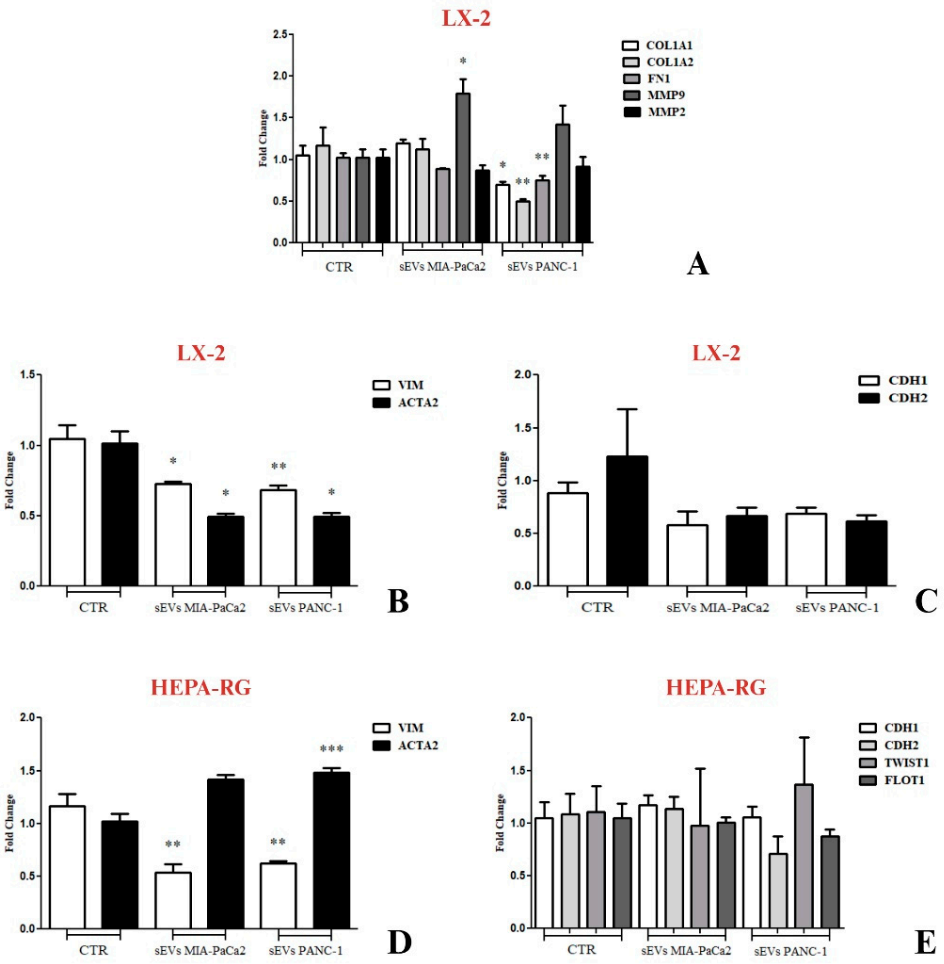

Table S3. **Clinicopathological characteristics of the PDAC patients**. Abbreviations: **G**, tumor grade; **TNM**, Tumor–Node–Metastasis staging system; **F**, female; **M**, male; **NA**, not available; **Mx**, distant metastasis not assessed.

Patient ID	Tumor Grade	TNM Stage	Sex	Age (years)
1	**G3**	**pT4N1Mx**	**F**	**68**
2	**G2**	**pT2N1Mx**	**M**	**66**
3	**G2**	**pT4N2Mx**	**M**	**48**
4	**G2**	**pT2N1Mx**	**M**	**55**

5	**G2**	**pT4N2Mx**	**M**	**66**
6	**G3**	**pT4N1Mx**	**F**	**76**
7	**G2**	**pT2N2Mx**	**F**	**50**
8	**G2**	**pT2N0Mx**	**F**	**53**
9	**G3**	**pT2N2Mx**	**M**	**81**
10	**G3**	**pT4N1Mx**	**M**	**70**
11	**G2**	**pT2N1Mx**	**F**	**55**
12	**NA**	**pT2N0Mx**	**M**	**68**
13	**G2**	**pT4N2Mx**	**F**	**44**
14	**G3**	**pT4N2Mx**	**M**	**81**
15	**G2**	**pT2N1Mx**	**F**	**79**
16	**G3**	**pT1cN1Mx**	**F**	**70**
17	**G3**	**pT4N1Mx**	**M**	**53**
18	**G2**	**pT1cN0Mx**	**F**	**70**
19	**G2**	**pT2N1Mx**	**M**	**58**
20	**G3**	**pT3N1Mx**	**F**	**63**
21	**G3**	**pT4N0Mx**	**M**	**54**
22	**G3**	**pT4N2Mx**	**F**	**66**
23	**G2**	**pT4N2Mx**	**F**	**77**
24	**G3**	**pT4N1Mx**	**M**	**77**
25	**G3**	**pT2N0Mx**	**F**	**76**

Figure S3. Effects of PDAC patient serum-derived sEVs on ECM remodelling and EMT marker expression in hepatic cell models. (A–C) qRT–PCR analysis in LX-2 cells treated with sEVs isolated from the serum of PDAC patients (sEVs PT). (A) Expression of ECM-related genes (COL1A1, COL1A2, FN1) and matrix remodeling enzymes (MMP9, MMP2). (B) Mesenchymal/activation markers VIM and ACTA2 (α-SMA). (C) Adhesion markers CDH1 (E-cadherin) and CDH2 (N-cadherin). (D–E) qRT–PCR analysis in HEPA-RG cells treated with PDAC patient-derived serum sEVs. (D) Expression of VIM and ACTA2. (E) Expression of CDH1, CDH2, TWIST1 (transcription factor), and FLOT1 (flotillin-1). Expression levels are shown as fold change relative to untreated control (CTR). Data represent mean ± SD from three independent experiments. Statistical significance was determined by one-way ANOVA followed by Dunnett's multiple comparisons test. *p < 0.05, **p < 0.01 vs CTR.

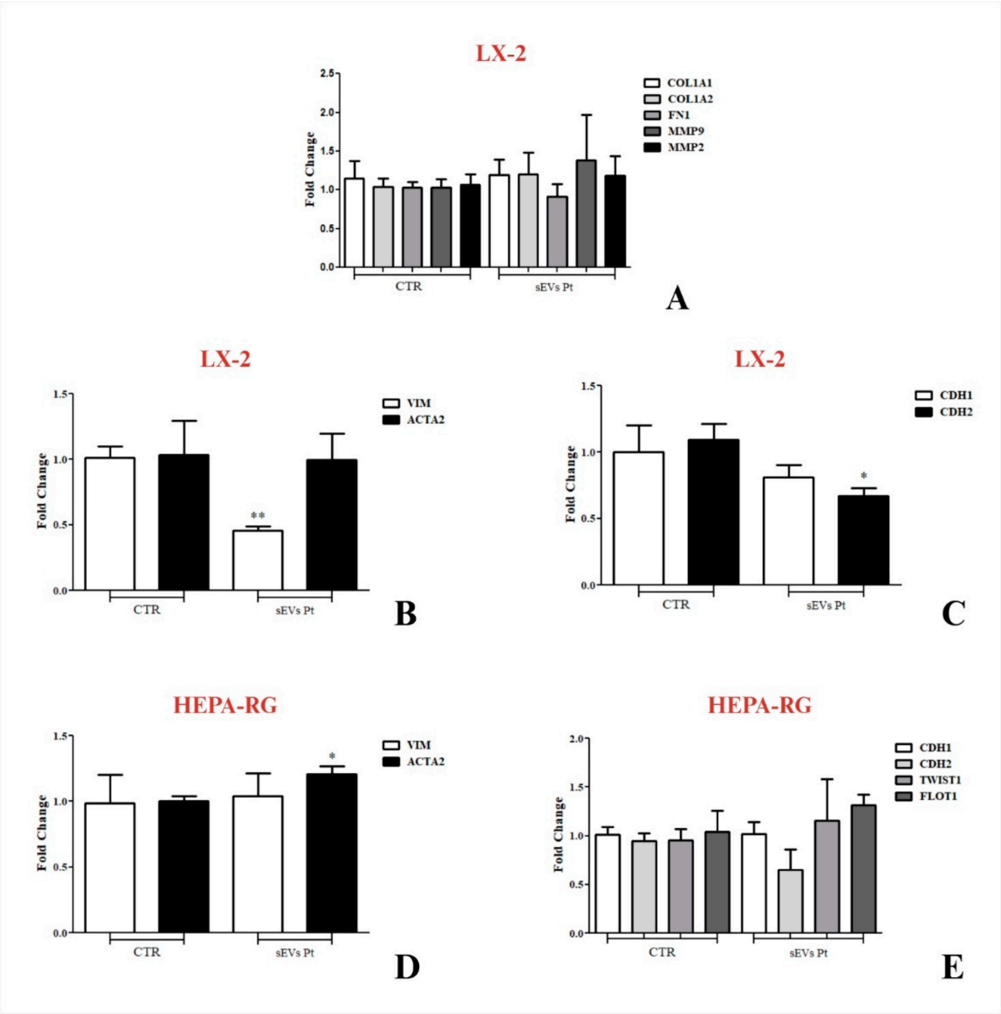

Supplement: Supplementary file 1 [file ijms-27-05270-s001.zip › ijms-4359064-supplementary.pdf]
